# Synergistic Control of *Bemisia tabaci* Using *Nesidiocoris tenuis* and *Orius laevigatus* and Its Effects on Tomato Yield

**DOI:** 10.3390/insects17060582

**Published:** 2026-06-03

**Authors:** Lassaad Mdallel, Abderrahman Mquitib, Abdallah Guerban, Bader Sulaiman Sudayri, Selman Al-Oudah, Soltan MMohamed Al-Eid

**Affiliations:** 1 National Organic Agriculture Center, Department of Protection and Biological Control, Ministry of Environment, Waiter and Agriculture, Unaiza 51911, Saudi Arabia; abdulrahman.mqt@gmail.com (A.M.); a25272011@gmail.com (A.G.); bss_882@hotmail.com (B.S.S.); aloudah.s@sofa.org.sa (S.A.-O.); 2 Saudi Organic Farming Association, Riyadh 11321, Saudi Arabia; e15637@mewa.gov.sa

**Keywords:** whitefly, biological control, greenhouse tomato, *Nesidiocoris tenuis*, *Orius laevigatus*, crop yield, fruit quality

## Abstract

The whitefly *Bemisia tabaci* is a major pest of greenhouse-grown tomato, causing significant yield and quality losses worldwide. This study evaluated the population dynamics of *B. tabaci* on tomato crops maintained at a maximum temperature of 24 ± 1 °C and assessed the effectiveness of two generalist predators, *Nesidiocoris tenuis* and *Orius laevigatus*, applied individually or in combination under greenhouse conditions in Saudi Arabia. Whitefly populations increased progressively throughout the study, reaching peak densities of 32.24 eggs and 124.00 ± 7.78 nymphs per leaf. Predator release significantly reduced *B. tabaci* populations at both the egg and nymphal stages. *N. tenuis* showed greater efficacy against eggs, achieving a 67.44% reduction, whereas *O. laevigatus* was slightly more effective against nymphs, with a 63.30% reduction. Notably, the combined release of both predators resulted in the greatest suppression of whitefly populations, reducing egg and nymphal densities by 79.50% and 78.02%, respectively, suggesting additive or synergistic interactions between the two predators. The dual-predator treatment also significantly improved yield-related parameters, including fruit number, fruit size, and total yield per plant, without adversely affecting fruit quality. In addition, vitamin C content increased under the combined predator treatment. These findings demonstrate that the integration of *N. tenuis* and *O. laevigatus* enhances biological control efficacy and supports sustainable integrated pest management strategies for greenhouse tomato production.

## Simple Summary

Whiteflies are among the most damaging pests in greenhouse tomato production, reducing both yield and fruit quality. In this study, we tested two beneficial insects, *Nesidiocoris tenuis* (Reuter 1895) and *Orius laevigatus* (Fieber, 1860), to control whiteflies. When used alone, each predator reduced whitefly numbers, but their combined use was much more effective. The dual release controlled both eggs and immature stages more efficiently and led to higher tomato yields without affecting fruit quality. This approach offers an environmentally friendly alternative to chemical insecticides and supports sustainable pest management in greenhouse systems.

## 1. Introduction

The tomato, *Solanum lycopersicum* L. (Solanaceae), is one of the most important cultivated crops worldwide [1,2,3]. In Saudi Arabia, it represents a major vegetable crop because of its high nutritional value and affordability. In 2020, the total cultivated area reached 12,454.3 ha, with a production of 351,212.4 tons of fresh fruit [3,4]. Tomato production requires carefully controlled environmental conditions to achieve optimal growth, flowering, fruit set, and yield. In greenhouse production systems, temperature and relative humidity are considered the most critical microclimatic factors affecting plant physiological processes and disease incidence. The optimal daytime temperature for tomato growth generally ranges from 22 to 26 °C, whereas nighttime temperatures should be maintained between 16 and 18 °C to ensure proper vegetative and reproductive development. Relative humidity (RH) in tomato greenhouses is ideally maintained between 60% and 70%, although acceptable ranges may vary from 50% to 85% depending on the growth stage and prevailing climatic conditions [5]. Tomato production is also constrained by several biotic factors, including fungal, bacterial, and viral pathogens, as well as nematodes and insect pests. Among these, insect pests are particularly important because they cause direct damage and may act as vectors of plant pathogens, resulting in significant reductions in yield and fruit quality [4,5,6,7,8,9,10,11].

Among insect pests, the whitefly *Bemisia tabaci* (Gennadius) (Hemiptera: Aleyrodidae) and the leaf miner *Tuta absoluta* (Meyrick) (Lepidoptera: Gelechiidae) are considered highly destructive species [12,13,14]. The whitefly, *B. tabaci*, causes severe damage to tomato plants by feeding on phloem sap, which inhibits plant growth, reduces yield, and facilitates the transmission of viral diseases. In addition, the excretion of honeydew promotes the development of sooty mold, thereby reducing photosynthetic efficiency and fruit quality [12,15,16,17,18].

For the management of the whitefly *B. tabaci*, farmers predominantly rely on chemical treatments to maintain populations below economic thresholds [19,20,21,22]. However, the intensive use of pesticides has detrimental effects on the environment and human health [23,24,25]. Moreover, repeated and large-scale applications have reduced their efficacy and contributed to the development of resistance in *B. tabaci* populations [26,27,28,29].

Given the increasing damage caused by *B. tabaci*, the global spread of insecticide resistance, and the need to mitigate the adverse effects of synthetic insecticides on beneficial organisms, applicators, consumers, and the environment, alternative pest management strategies are urgently required. Among these, the use of natural enemies represents a promising and sustainable approach for the control of whiteflies on tomato and other crops [16,26,30,31,32,33]. To date, several studies have demonstrated that a diverse array of natural enemy predators including members of the families Coccinellidae (e.g., *Delphastus pusillus* LeConte), Miridae (*Macrolophus pygmaeus* Reuter; *Nesidiocoris tenuis* Reuter), and Anthocoridae (*Orius albidipennis* Reuter; *Orius laevigatus* Fieber) actively prey on *B. tabaci* across a wide range of crops [34,35,36]. In addition, several parasitoid species belonging to the family Aphelinidae, such as *Encarsia formosa* Gahan, *Eretmocerus eremicus* Rose and Zolnerowich, and *Eretmocerus mundus* Mercet, have been widely investigated for their effectiveness against *B. tabaci* [37,38,39]. Furthermore, several entomopathogenic fungi, including *Beauveria bassiana* (Balsamo-Crivelli) Vuillemin, *Cordyceps fumosorosea* (Wize) Kepler, *Beauveria shrestha* (Brown and G. Smith), and *Isaria fumosorosea* (Wize), have been extensively investigated as biological control agents against *B. tabaci* [40,41].

Several studies have also examined the use of natural enemies, particularly the predators *N. tenuis* and *O. laevigatus*, released individually to control *B. tabaci* on various crops under greenhouse conditions [16,34,42]. For instance, Calvo et al. [16] reported that releasing *N. tenuis* at a density of 1–4 individuals per plant reduced whitefly populations by up to 90% in tomato crops. In a subsequent study, Calvo et al. [43] demonstrated that *N. tenuis* effectively controlled both *Tuta absoluta* and *B. tabaci*, either alone or in combination with other agents. Similarly, Assadi et al. [44] found that introducing *N. tenuis* at densities of 1–4 nymphs per tobacco plant significantly reduced *B. tabaci* populations, with efficacy reaching up to 98% against both nymphal and adult stages.

Regarding *O. laevigatus*, Salama et al. [42] showed that this predator can feed on all developmental stages of *B. tabaci* and successfully complete its life cycle when provided exclusively with whitefly eggs, nymphs, and adults. The authors further reported that female *O. laevigatus* consumed higher numbers of eggs, fourth-instar nymphs, and adults compared to earlier nymphal instars across different prey densities. In addition, Arno et al. [45] identified *O. laevigatus* as an effective biological control agent against *B. tabaci*.

The use of combined biological control agents has also been widely investigated [16,35,46,47]. For example, Calvo et al. [16] demonstrated that combining *Encarsia mundus* with either *Amblyseius swirskii* Athias-Henriot or *Macrolophus caliginosus* Wagner significantly reduced whitefly populations on sweet pepper and tomato crops. Similarly, Vafaie et al. [48] showed that the combined release of *E. eremicus* and *A. swirskii* is a feasible strategy for managing *B. tabaci*, reducing insecticide applications by 25–78%. In cucumber crops, Adly et al. [35] reported that a combination of *Chrysoperla carnea* (Steph.), *Orius albidipennis* (Reuter), and *Phytoseiulus persimilis* Athias-Henriot provided effective control of *B. tabaci*. Heinz et al. [49] further demonstrated that the combined release of the predator *Delphastus pusillus* (LeConte) and parasitoids of the genus *Encarsia* resulted in greater suppression of whitefly populations compared to parasitoids alone. Likewise, Gabara et al. [50] reported that the most effective control of *B. tabaci* in greenhouse tomato crops was achieved through the combined use of the parasitoid *E. mundus* and the predator *M. caliginosus*.

Potential synergistic interactions among biological control agents may vary depending on the timing and strategy of their release. Under simultaneous release, predators and parasitoids can complement each other by attacking different developmental stages of *B. tabaci*, resulting in rapid population suppression [16,49]. In contrast, staggered or sequential releases may enhance long-term control by allowing parasitoids to establish first, followed by predators that suppress surviving individuals and prevent pest resurgence while minimizing intraguild interference [48,50].

Despite recent advances, no previous studies have specifically assessed the combined efficacy of *N. tenuis* and *O. laevigatus* in suppressing *B. tabaci* in organic greenhouse tomato systems, nor their impact on production quality. The presence of these predators in such systems may be particularly important for the simultaneous management of multiple pests, including *T. absoluta*, mites, and whiteflies, as well as for maintaining crop quality.

In the present study, we evaluated the efficacy of the two predatory hemipterans, *O. laevigatus* (i) and *N. tenuis* (i), in controlling *B. tabaci* in organic greenhouse tomato crops when released either individually or in combination (ii), and their effects on production quality. We hypothesized that (i) each predator would significantly reduce *B. tabaci* populations under the environmental and crop-management conditions of the present study, including the specific greenhouse conditions, predator release rates, and prey densities evaluated, and (ii) their combined release would result in enhanced suppression and improved production quality.

## 2. Materials and Methods

### 2.1. Greenhouse Experiment

The experiment was conducted at the National Organic Agriculture Center in Unaizah, Al-Qassim, Saudi Arabia (26.085478° N, 43.9768123° E), from December 2024 to June 2025. The study was carried out in a 360 m^2^ greenhouse, which was divided longitudinally into two sections. Prior to planting, the soil in each section was prepared into three ridges, each 36 m in length and spaced 1 m apart. Each section was further subdivided into nine plots, each measuring 12 m^2^. All plots were completely enclosed with insect-proof netting to prevent the movement of whiteflies and their natural enemies between treatments.

Tomato seedlings (cv. Newton), obtained from a commercial nursery in Unaizah, were certified free of pests and diseases prior to transplanting. Seedlings were transplanted on 15 December 2024 in a greenhouse equipped with a drip irrigation system. Plants were spaced at 50 cm intervals within plots, and each experimental plot consisted of nine plants, giving 162 plants across all treatments. Greenhouse conditions were maintained at 24 ± 1 °C with a relative humidity of 60–70% under solar radiation throughout the experimental period. From transplanting until the onset of fruiting, plants received weekly fertigation with neutral NPK fertilizers (4–12–5, 7–5–4, and 5–5–14) at the manufacturer’s recommended rates.

### 2.2. Experimental Design

The experiment was arranged in a randomized complete block design (RCBD) with three replications and six treatments. The treatments were as follows: (1) single release of *N. tenuis*, (2) single release of *O. laevigatus*, (3) combined release of *N. tenuis* and *O. laevigatus*, (4) untreated control, (5) organic insecticide Azdar (active ingredient: azadirachtin), and (6) chemical insecticide dinotefuran 20% SG. A total of 162 tomato plants were used in the experiment. Each treatment included 27 plants, arranged as nine plants per plot across the three replications.

### 2.3. Development of B. tabaci Eggs and Numphae on Tomato Crops

*B. tabaci* newly emerged individual adults were collected from tomato plants in a separate greenhouse and released into each experimental plot in early February 2025 (when plant at age of approximately 2 months) at a rate of 20 adults per plot. Plants in Treatment 4 (untreated control) were monitored weekly until 30 May 2025. For each plant within a plot, three leaves were randomly selected, and the total number of larvae and eggs per leaf was recorded. Counts were performed in situ using a DM Wi-Fi digital microscope (TOMLOV, Shenzhen, China) (1000×; Ver. 1.9, build 21) without detaching the leaves.

### 2.4. Experiment Execution and Recorded Data

On 18 March 2025, when the plants were 3 months old, adult predators (*O. laevigatus* and *N. tenuis*) were obtained from the Biocontrol and Bumblebee Production Center (Unaizah, Al-Qassim, Saudi Arabia) and released into the plots within 12 h of arrival. Two hours prior to predator release, three leaves were randomly selected from each plant within each plot, and the number of *Bemisia tabaci* larvae per leaf was counted using a DM Wi-Fi digital microscope (1000×; Ver. 1.9, Build 21). Predator releases were conducted on two occasions (18 March and 20 April 2025) across six treatments: (1) *O. laevigatus* released at 1 predator m^−2^; (2) *N. tenuis* released at 1 predator m^−2^; (3) a combined release of *O. laevigatus* and *N. tenuis* at 0.5 + 0.5 predators m^−2^; (4) an untreated control; (5) an organic insecticide treatment using Azdar applied at 2 mL L^−1^; and (6) a chemical insecticide treatment using Dominate applied at 0.5 mL L^−1^. All treatments were applied simultaneously. The chemical and organic insecticide applications were repeated at 15-day intervals until the end of the experiment.

Regular data collection began seven days after the initial treatment and continued weekly until 30 May 2025, following the same leaf-sampling and counting procedures, conducted two hours prior to each release. The effectiveness of *O. laevigatus*, *N. tenuis*, their combination, Azdar, and Dominate was evaluated. Efficacy was calculated as the percentage reduction in *B. tabaci* larvae or eggs using the following formula:

Efficacy (%) = [(NC_dn1_ − NT_dn1_)/(NC_dn1_)] × 100

where NC_dn1_ is the number of *B. tabaci* larvae or egg per leaf on untreated control plants, NT_dn1_ is the number of larvae or egg per leaf after application of given treatment, and d represents the weekly sampling.

### 2.5. Effects of Treatments on Tomato Yield and Fruit Nutritional Quality

All harvested tomato fruits from each treatment were collected to determine yield and its components, including the number of fruits per plant, average fruit weight (g), fruit diameter (cm), and total fruit yield per plant (g). Representative samples (1 kg of fruits per treatment) were collected and transferred to the Central Laboratory of the National Organic Agriculture Center for the evaluation of fruit quality attributes, including total acidity, protein content, vitamin C, total soluble phenols, and total soluble solids.

Total acidity of tomato samples was determined and expressed as citric acid according to AOAC (17th ed., 2000) [51]. A 10 g aliquot of filtered tomato juice was transferred into a 100 mL volumetric flask, and 25 mL of distilled water was added. Phenolphthalein was used as an indicator to detect the titration endpoint. The sample was titrated with 0.1 N sodium hydroxide until a persistent color change was observed. Total acidity (%) was calculated using the following equation:

Acidity (%) = (N × V × M)/(S × 10)

where N is the normality of the sodium hydroxide solution, V is the volume (mL) of sodium hydroxide used, M is the molecular weight of the predominant acid (citric acid) divided by the number of ionizable hydrogen atoms, and S is the sample weight (g) or volume (mL).

Nitrogen content of the tomato samples was determined after drying in a hot-air oven at 70 °C for 24 h using the Kjeldahl method. The nitrogen content was then converted to crude protein using a factor of 6.25. The Kjeldahl method involves the conversion of organic nitrogen into ammonium sulfate during digestion with concentrated sulfuric acid in the presence of copper sulfate and potassium sulfate as catalysts. The digested sample was distilled using a Kjeldahl distillation unit (VELP) to release ammonia, which was captured in 4% boric acid solution and subsequently titrated with 0.1 N hydrochloric acid. The nitrogen content was calculated and expressed as protein percentage.

Total phenolic content (TPC) was determined spectrophotometrically using the Folin–Ciocalteu reagent. An aliquot of the sample extract was mixed with the reagent, followed by the addition of sodium carbonate solution to develop color. The mixture was incubated at room temperature for 1 h, resulting in the formation of a blue-colored complex. Absorbance was measured at 765 nm using a double-beam spectrophotometer. TPC was quantified using a calibration curve prepared with gallic acid and expressed as mg gallic acid equivalents (GAE) per unit weight of the sample [52].

Total soluble solids (TSS) were measured using a manual refractometer at 20 °C and expressed as °Brix. Vitamin C content was determined using an HPLC system (Thermo Ultimate 3000, Waltham, MA, USA) according to Campos et al. [53].

Mineral contents were determined in dried, ground samples. A 0.5 g portion of each sample was transferred into a Teflon digestion vessel, and 5 mL of H_2_SO_4_ and 1 mL of H_2_O_2_ were added. After microwave digestion, the solution was transferred into a 100 mL volumetric flask and diluted to volume with deionized water. Mineral concentrations (mg/kg) were measured using inductively coupled plasma optical emission spectroscopy (ICP-OES; ICAP 7200 DUO, Thermo Fisher Scientific, Waltham, MA, USA.) [54].

Phosphorus content was determined spectrophotometrically using the ammonium molybdate method, which is based on the formation of a phosphomolybdate complex. This complex was reduced (commonly using ascorbic acid) to form a blue-colored compound (molybdenum blue), and absorbance was measured at 880 nm using a PD-303UV spectrophotometer (Kawaguchi, Saitama, Japan) [55].

Potassium and calcium contents were determined using a flame photometer (BWB Technologies Ltd., Hambridge Ln, Newbury, UK), and the results were expressed in mg/kg of the sample.

### 2.6. Data Analysis

The effectiveness data were analyzed using one-way analysis of variance (ANOVA). When significant differences were detected, treatment means were compared using Tukey’s post hoc test. All statistical analyses were conducted using SPSS software (version 22; IBM Corp., Armonk, NY, USA). Differences were considered statistically significant at *p* ≤ 0.05.

## 3. Results

### 3.1. Evolution of B. tabaci Eggs and Numphae on Tomato Plants

**Figure 1:**
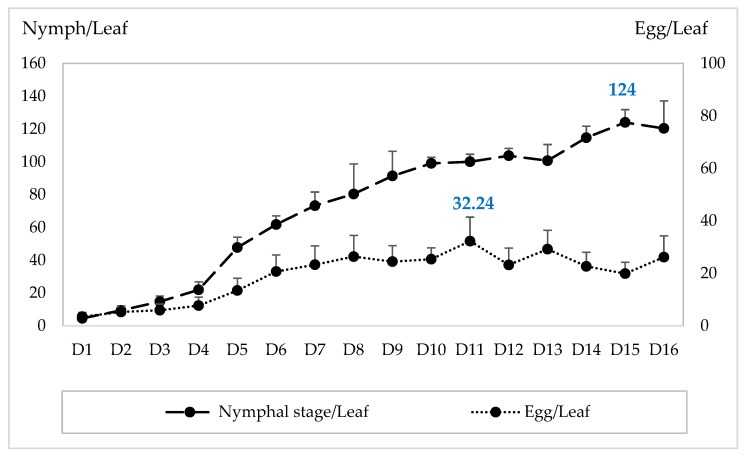
Weekly counts of eggs and nymphae of *B. tabaci* per leaf of tomato plant in the untreated plots during the experiment. (D1–D16: Weekly counts of eggs and numphae per tomato leaf from the first sampling event (D1) to the end of experiment (D16)).

Egg and nymph densities of *B. tabaci* per tomato leaf in the untreated plots were monitored weekly from 10 February to 30 May 2025. The temporal dynamics of egg and nymph densities are presented in Figure 1. Egg densities showed a progressive increase over the 16-week observation period (112 days), with mean values ranging from 3.44 ± 1.66 to 32.24 ± 9.16 eggs per leaf. The highest egg density (32.24 ± 9.16) was recorded at D11. Similarly, nymph densities varied between 4.48 ± 2.11 and 124 ± 7.78 individuals per leaf. The peak nymph density (124 ± 7.78) was observed at D15.

### 3.2. Impact of Treatments on B. tabaci Eggs on Tomato Plants

**Figure 2:**
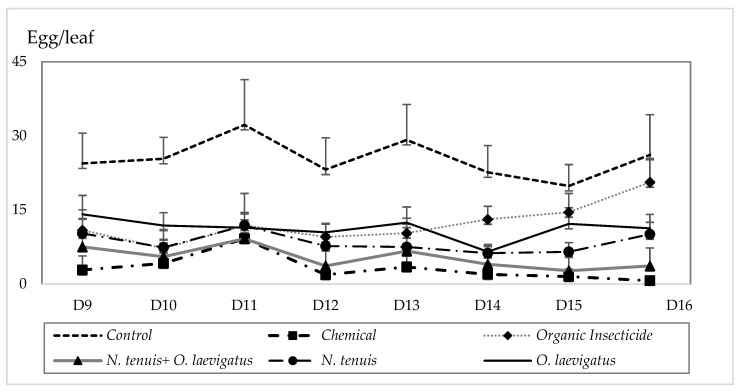
Counts of *B. tabaci* eggs on tomato plants under different treatments. (D9–D16: weekly count after application of the treatments).

Figure 2 shows the number of *B. tabaci* eggs per tomato leaf under greenhouse conditions across the different treatments. During the treatment period (D9–D16), the weekly mean number of eggs per leaf ranged from 0.68 ± 0.22 to 32.24 ± 9.16. The highest mean number of eggs (32.24 ± 9.16) was recorded on D11 in the untreated control. In contrast, the lowest mean number of eggs (0.68 ± 0.22) was observed on D16 in the chemical treatment. In the organic insecticide treatment, the mean number of eggs ranged from 7.25 ± 1.64 on D10 to 20.61 ± 4.80 on D16. Following the combined release of *O.laevigatus* and *N. tenuis*, the mean number of eggs ranged from 2.69 ± 0.44 on D15 to 9.18 ± 1.26 on D11. After the single release of *N. tenuis*, the lowest mean number of eggs (6.24 ± 1.33) was recorded on D14. Similarly, after the single release of *O. laevigatus*, the lowest mean number of eggs (6.54 ± 1.24) was also recorded on D14. Statistical analysis revealed highly significant differences (*p <* 0.001) between the chemical treatment and the combined release treatment compared with the single release treatments, the organic insecticide treatment, and the untreated control at D9, D10, D11, D12, D13, D14, D15 and D16.

**Figure 3:**
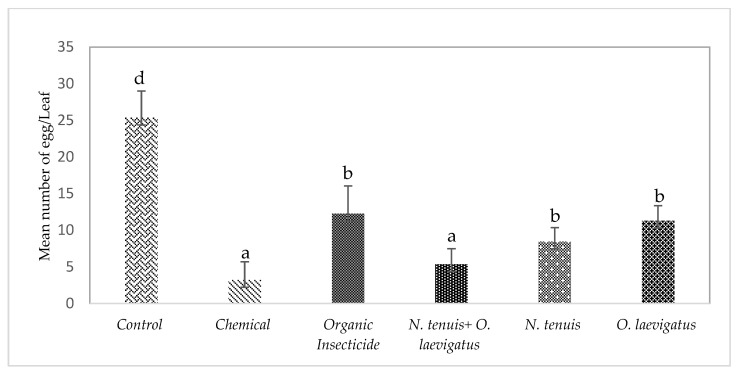
Mean number of *B. tabaci* egg per leaf under different treatments. Data are presented as mean ± standard error (SE). Different lowercase letters indicate statistically significant differences among treatments according to Tukey’s honestly significant difference (HSD) test at *p* < 0.05.

The mean number of *B. tabaci* eggs per tomato leaf during the D9–D16 period is presented in Figure 3. The highest mean number of eggs (25.37 ± 3.63 eggs leaf^−1^) was recorded in the untreated control treatment, whereas the lowest mean number (3.21 ± 2.48 eggs leaf^−1^) was observed under the chemical treatment. The combined release of *O. laevigatus* and *N. tenuis* resulted in a mean of 5.36 ± 2.11 eggs leaf^−1^. In contrast, mean egg numbers of 8.44 ± 1.89, 11.28 ± 2.05, and 12.25 ± 3.77 eggs leaf^−1^ were recorded following the single release of *N. tenuis*, the single release of *O. laevigatus*, and the organic insecticide treatment, respectively. Statistical analysis revealed highly significant differences (*p* = 0.000) between the chemical treatment and the combined release treatment compared with the single release treatments, the organic insecticide treatment, and the untreated control.

**Figure 4:**
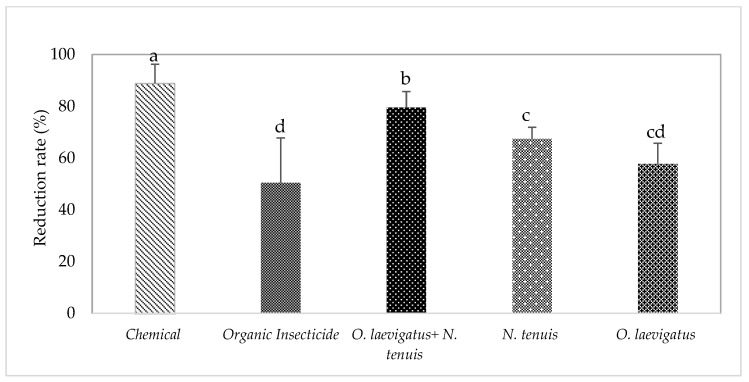
Reduction in the mean density of eggs of *B. tabaci* per tomato leaf under different treatments. Data are presented as mean ± standard error (SE). Different lowercase letters indicate statistically significant differences among treatments according to Tukey’s honestly significant difference (HSD) test at *p* < 0.05.

Figure 4 illustrates the reduction rates in egg density of *B. tabaci* on tomato leaves under different treatments. Reduction rates ranged from 50.47 ± 17.27 to 88.81 ± 7.39%. The highest reduction (88.81 ± 7.39%) was recorded for the chemical treatment, whereas the lowest (50.47 ± 17.27%) was observed with the organic insecticide. The release of *O. laevigatus* resulted in a reduction rate of 57.71 ± 7.99%, while a single release of *N. tenuis* achieved a reduction of 67.44 ± 4.41%. The combined release of *N. tenuis* and *O. laevigatus* led to a higher reduction rate of 79.50 ± 6.17%. A highly significant difference in reduction rates was detected among treatments (ANOVA: F = 17.98, df = 4, *p* < 0.001).

### 3.3. Impact of Treatments on B. tabaci Numphal Stages on Tomato Plants

**Figure 5:**
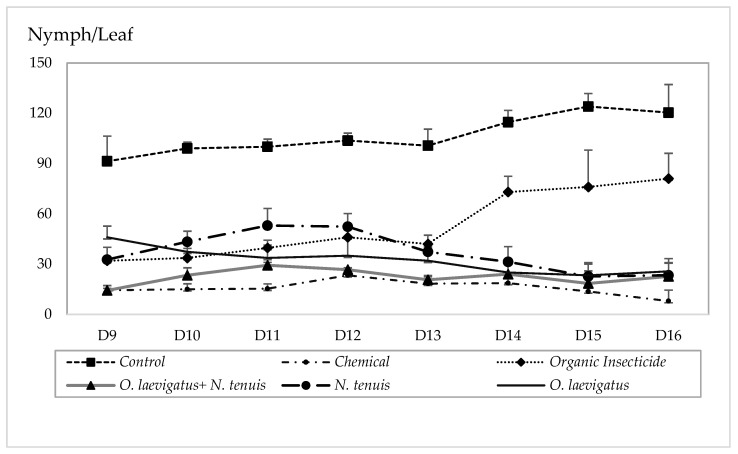
Counts of *B. tabaci* nymphs on tomato plants under different treatments. (D9–D16: weekly count after application of the treatments).

Figure 5 illustrates the mean number of *B. tabaci* nymphs per tomato leaf under greenhouse conditions across the different treatments. During the D9–D16 period, the weekly mean number of nymphs per leaf ranged from 8.00 ± 6.53 to 120.33 ± 16.81. The highest mean number of nymphs (120.33 ± 16.81) was recorded in the untreated control on D16, whereas the lowest mean number (8.00 ± 6.53) was observed in the chemical treatment on the same date. In the organic insecticide treatment, the mean number of nymphs increased from 32.00 ± 4.70 on D9 to 81.00 ± 15.12 on D16. Following the combined release of *O. laevigatus* and *N. tenuis*, the mean number of nymphs ranged from 14.33 ± 2.86 on D9 to 29.33 ± 1.69 on D11. In the single-release treatment of *N. tenuis*, the lowest mean number of nymphs (22.66 ± 3.29) was recorded on D15. Similarly, in the single-release treatment of *O. laevigatus*, the lowest mean number of nymphs (23.33 ± 7.58) was also observed on D15. Statistical analysis revealed highly significant differences (*p* < 0.001) between the chemical treatment compared with the combined release treatment, the single release treatments, the organic insecticide treatment, and the untreated control on D9, D10, D11, D12, D13, D14, D15 and D16.

**Figure 6:**
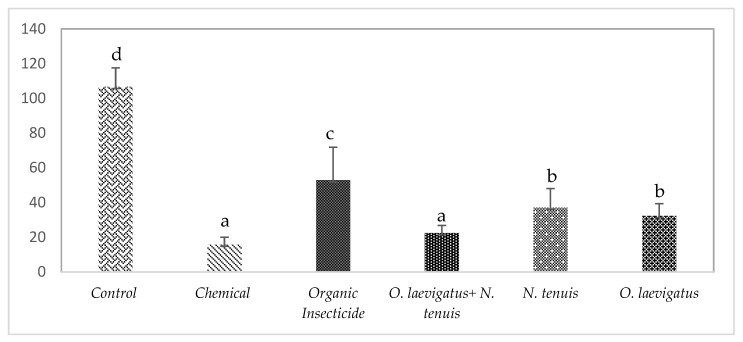
Mean number of *B. tabaci* nymphs per leaf under different treatments. Data are presented as mean ± standard error (SE). Different lowercase letters indicate statistically significant differences among treatments according to Tukey’s honestly significant difference (HSD) test at *p* < 0.05.

During the D9–D16 assessment period, significant variation in the mean density of *B. tabaci* nymphs per tomato leaf was observed among treatments (Figure 6). The untreated control exhibited the highest infestation level, with a mean of 106.7 ± 10.80 nymphs leaf^−1^, whereas the chemical treatment achieved the greatest suppression, recording the lowest mean density (15.83 ± 4.17 nymphs leaf^−1^). The combined release of *O. laevigatus* and *N. tenuis* also markedly reduced nymph populations, resulting in a mean of 22.42 ± 4.38 nymphs leaf^−1^. In comparison, the individual release of *N. tenuis* and *O. laevigatus* yielded mean densities of 37.00 ± 11.03 and 32.24 ± 7.07 nymphs leaf^−1^, respectively, while the organic insecticide treatment recorded 52.91 ± 18.95 nymphs leaf^−1^. Statistical analysis demonstrated highly significant differences among treatments (*p* < 0.001), with both the chemical treatment and the combined predator release showing superior efficacy relative to the single-predator treatments, the organic insecticide treatment, and the untreated control.

**Figure 7:**
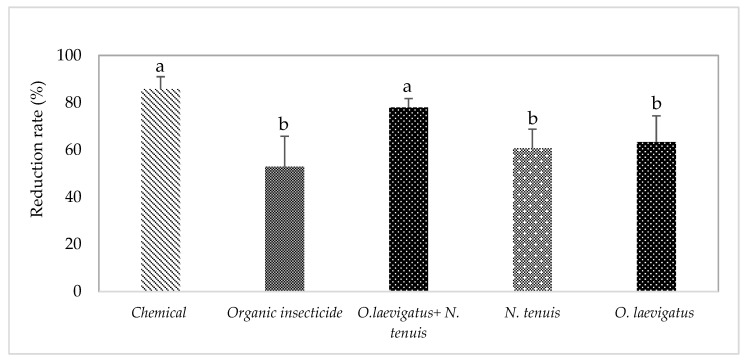
Reduction in the mean density of numphae of *B. tabaci* per tomato leaf under different treatments. Data are presented as mean ± standard error (SE). Different lowercase letters indicate statistically significant differences among treatments according to Tukey’s honestly significant difference (HSD) test at *p* < 0.05.

Figure 7 shows the reduction in density of *B. tabaci* nymphs on tomato leaves under different treatments. Reduction rates ranged from 52.88 ± 12.86% to 85.74 ± 5.22%. The highest reduction (85.74 ± 5.22%) was recorded for the chemical treatment, whereas the lowest (52.88 ± 12.86%) was observed with the organic insecticide. The release of *O. laevigatus* resulted in a reduction of 63.30 ± 11.12%, while a single release of *N. tenuis* achieved a reduction of 60.73 ± 8.07%. In contrast, the combined release of *N. tenuis* and *O. laevigatus* led to a higher reduction rate of 78.02 ± 3.17%. Statistical analysis revealed a highly significant difference among treatments (F = 14.19, df = 4, *p* < 0.001).

### 3.4. Efficacy of N. tenuis and O. laevigatus to Control B. tabaci on Tomato Plant

The reduction rates of *B. tabaci* egg and nymph densities per tomato leaf following single and combined releases of *N. tenuis* and *O. laevigatus* are presented in Figure 4 and Figure 7. The results indicate that *N. tenuis* exhibited a greater capacity to reduce egg densities of *B. tabaci*, achieving a mean reduction rate of 67.44 ± 4.41%, compared with 57.71 ± 7.99% for *O. laevigatus*. Notably, the combined release of both predator species resulted in a substantially higher reduction rate of *B. tabaci* eggs (79.50 ± 6.17%). Statistical analysis revealed that predator treatment had a highly significant effect on egg reduction rates (ANOVA: F = 20.59; df = 2; *p* < 0.001). In contrast, *O. laevigatus* demonstrated a greater capacity to reduce nymph densities of *B. tabaci*, with a mean reduction rate of 63.30 ± 11.12%, compared with 60.73 ± 8.07% for *N. tenuis*. Similarly, the combined release of both predators resulted in a markedly higher reduction rate of *B. tabaci* nymphs (78.02 ± 3.17%). Statistical analysis confirmed that predator treatment also had a highly significant effect on nymph reduction rates (ANOVA: F = 8.01; df = 2; *p* = 0.003).

### 3.5. Effects of Treatments on Tomato Yield and Fruit Nutritional Quality

Data presented in Table 1 indicate that both single and combined releases of *N. tenuis* and *O. laevigatus* improved tomato yield components compared with the untreated control. The highest fruit number per plant (79.89), fruit diameter (21.72 cm), fruit weight (138.66 g), and total fruit yield per plant (11,060 g) were recorded under chemical treatment. Among the biological control treatments, the combined release of the two predators resulted in the highest fruit number per plant (76.15), fruit diameter (20.88 cm), fruit weight (131.44 g), and total fruit yield per plant (10,090 g). Statistical analysis revealed significant differences among treatments for fruit number per plant (ANOVA: F = 8.49; df = 5; *p* = 0.001) and total fruit yield per plant (ANOVA: F = 10.96; df = 5; *p* < 0.001). However, no significant differences were detected among treatments for fruit weight (*p* = 0.23) or fruit diameter (*p* = 0.13).

With respect to tomato fruit quality, the data presented in Table 2 indicate that the highest nitrogen (2.4%) and protein (13.97%) contents were recorded under the combined release of *N. tenuis* and *O. laevigatus*. In contrast, phosphorus content was slightly reduced under the combined release compared with the other treatments; however, these differences were not statistically significant (*p* = 0.901). Similarly, potassium concentration showed an increasing trend under the combined release, although no significant differences were detected among treatments (*p* = 0.427). The lowest values of total phenols (0.32) and total dissolved solids (TDS, 0.50%) were also observed under the combined release, with no significant differences (*p* = 0.96 and *p* = 0.26, respectively). Furthermore, the highest vitamin C content (124.66 mg·100 g^−1^) was associated with the combined release treatment, this increase was statistically significant compared to the other treatments (*p* = 0.022).

## 4. Discussion

The whitefly, *B. tabaci*, is one of the most destructive insect pests affecting greenhouse-grown tomato and numerous other crops, causing substantial reductions in yield and fruit quality [26,56]. Extensive research has addressed its biology, population dynamics, seasonal abundance, and the extent of damage it causes in tomato systems [57,58,59].

In the present study, the densities of *B. tabaci* eggs and nymphs per tomato leaf increased progressively throughout the observation period, reaching maximum values of 32.24 eggs per leaf and 124 ± 7.78 nymphs per leaf. This upward trend and the relatively high infestation levels can likely be attributed to a combination of interacting biotic and abiotic factors, including temperature, host plant chemistry, amino acid composition, and overall nutritional quality [60,61,62]. Leaf morphological traits have been shown to significantly influence whitefly infestation. For example, Žanić et al. [62] reported a negative correlation between leaf thickness and both egg and nymph densities, suggesting that thicker leaves may act as a mechanical barrier to oviposition and feeding. Temperature is another key abiotic factor regulating the development, survival, and population growth of *B. tabaci* [63]. Sani et al. [30] demonstrated that the developmental rate of *B. tabaci* follows a nonlinear response to temperature, with optimal performance occurring between 20 and 33 °C and peak fecundity observed at approximately 25–28 °C. Therefore, the population increase observed in this study likely reflects favorable environmental conditions combined with suitable host plant characteristics under greenhouse conditions.

Natural enemies play a fundamental role in the management of *B. tabaci*, particularly parasitoids such as *E. formosa*, *E. eremicus*, and *E. mundus*, as well as generalist predators including *N. tenuis* and *O. laevigatus* [16,34,37,38,39,42]. These biological control agents have been widely recognized as key components of integrated pest management (IPM) programs for suppressing whitefly populations under both greenhouse and open-field conditions.

The present study demonstrated that single releases of *N. tenuis* and *O. laevigatus* significantly reduced egg densities of *B. tabaci*. Between the two predators, *N. tenuis* exhibited greater predation efficiency against eggs, achieving a mean reduction of 67.44%, compared with 57.71% for *O. laevigatus*. However, the combined release of both predator species resulted in the highest suppression level (79.50%), indicating a possible additive or synergistic interaction between the two predators. A similar trend was observed for the nymphal stage, where *O. laevigatus* showed slightly higher efficacy (63.30%) than *N. tenuis* (60.73%). Nevertheless, the combined treatment achieved the greatest reduction in nymphal populations (78.02%). These findings highlight the complementary roles of both predators in targeting multiple developmental stages of the pest and support their combined use in biological control programs against *B. tabaci*.

The superior performance of *N. tenuis* against whitefly eggs may be attributed to its zoophytophagous feeding behavior, which enables it to utilize both plant and prey resources. This ecological flexibility enhances predator establishment, persistence, and predation capacity under varying environmental conditions [42,64]. In addition, this mirid predator is known to suppress several other pests, including aphids and lepidopteran eggs, while also inducing plant defense responses through phytophagy [16,64]. In contrast, *O. laevigatus* is primarily recognized as a predator of thrips, although it can also feed on whitefly eggs and early instars, generally with lower efficiency [45,65]. The present results are consistent with previous studies demonstrating the effectiveness of mirid predators, particularly *N. tenuis*, in suppressing *B. tabaci* populations in greenhouse crops [66]. Similarly, *O. laevigatus* has been considered an important complementary component of IPM programs rather than a primary whitefly control agent [45].

Regarding tomato yield and fruit quality, the present findings clearly showed that the combined release of *N. tenuis* and *O. laevigatus* significantly improved yield components compared with single-predator releases, organic insecticide treatments, and untreated controls. The dual-predator treatment produced the highest number of fruits per plant (76.15), fruit diameter (20.88 cm), average fruit weight (131.44 g), and total yield per plant (10,090 g). Furthermore, this treatment maintained similar levels of nitrogen, phosphorus, potassium, and protein content, while increasing vitamin C concentration relative to the other treatments.

The improved crop performance observed under the combined predator treatment may be explained by complementary feeding behavior and niche partitioning between the two predators, which likely enhanced suppression of a broader spectrum of pest species. *N. tenuis* is capable of controlling multiple pests, including whiteflies, aphids, and lepidopteran eggs, whereas *O. laevigatus* primarily targets thrips and other small arthropods, thereby reducing pest pressure during critical stages of fruit development [67,68]. Consequently, the integration of these predators may have enhanced plant health and improved resource allocation toward fruit production. These findings are in agreement with earlier studies highlighting the benefits of combining generalist predators within IPM programs [45,66].

Moreover, the present study supports the growing evidence that biological control strategies can provide sustainable and environmentally friendly alternatives to conventional insecticide-based programs while maintaining or even enhancing crop productivity. From an economic perspective, the combined use of *N. tenuis* and *O. laevigatus* represents a cost-effective approach over the long term, despite the relatively higher initial investment associated with predator acquisition and release. Sustained suppression of *B. tabaci* populations can substantially reduce insecticide applications, labor costs, and crop losses associated with whitefly infestations. In contrast, intensive chemical control programs often increase production costs due to repeated pesticide applications and the development of insecticide resistance in whitefly populations. Additionally, biological control agents contribute to the conservation of beneficial arthropods and environmental sustainability, which are increasingly important in protected tomato production systems. Nevertheless, the success of predator-based strategies depends on their proper integration within IPM programs and careful monitoring of predator populations to avoid potential phytophagous damage caused by excessive densities of *N. tenuis* [44,69].

## 5. Conclusions

In conclusion, the present study demonstrates that *B. tabaci* populations can reach high densities under favorable greenhouse conditions, driven by environmental factors and host plant characteristics. The results clearly show that both *N. tenuis* and *O. laevigatus* are effective biological control agents; however, their combined application provided significantly greater suppression of both egg and nymph stages, indicating a synergistic interaction. Moreover, the dual-predator strategy not only enhanced pest control but also significantly improved tomato yield and maintained fruit quality, highlighting its practical value in greenhouse production systems. These findings emphasize the importance of predator diversity and support the incorporation of multiple natural enemies into integrated pest management programs as a sustainable alternative to chemical control. In addition, the successful implementation of this dual-predator approach may contribute to reducing reliance on chemical insecticides, thereby minimizing risks associated with pesticide resistance, environmental contamination, and adverse effects on non-target organisms, while promoting safer and more sustainable greenhouse tomato production.

## Figures and Tables

**Table 1:** Effects of different treatments targeting *B. tabaci* on tomato yield and components under controlled greenhouse conditions.

Treatment	Fruit/Plant	Fruit Diameter (cm)	Fruit Weight (g)	Fruit Yield/Plant (g)
Control	41.71 ± 1.49 ^c^	20.44 ± 1.01	125.00 ± 15.23	5210 ± 1870 ^c^
Organic insecticide	68.34 ± 2.98 ^b^	20.55 ± 1.21	128.22 ± 19.56	8760 ± 1260 ^b^
*O. leavegatus*	59.1 ± 1.97 ^bc^	19.83 ± 1.22	113.00 ± 19.36	6670 ± 2230 ^c^
*N. tenuis*	74.87 ± 8.11 ^a^	20.83 ± 1.54	128.55 ± 23.81	9620 ± 1040 ^b^
*O. laevigatus* + *N. tenuis*	76.15 ± 4.40 ^a^	20.88 ± 1.30	131.44 ± 20.05	10,090 ± 570 ^a^
Chemical insecticide	79.89 ± 13.93 ^a^	21.72 ± 1.49	138.66 ± 21.39	11,060 ± 1940 ^a^

Different lowercase letters indicate statistically significant differences among treatments according to Tukey’s honestly significant difference (HSD) test at *p* < 0.05.

**Table 2:** Effects of different treatments targeting *B. tabaci* on tomato fruit quality under controlled greenhouse conditions.

Treatment	Nitrogen (%)	Phosphorus (%)	Potassium (%)	Protein (%)	Vitamin C (mg/100 g)	Total Acidity (g Citric Acid/100 g Fruit)	Total Phenol (mg/100 g)	Total Dissolved Soluble (%)
Control	1.79 ± 0.20	4.86 ± 0.49	2.1 ± 0.20	12.12 ± 2.15	91.80 ± 13.1 ^c^	0.36 ± 0.008	0.32 ± 0.17	0.55 ± 0.032
Organic insecticide	1.98 ± 0.11	5.13 ± 1.76	2.3 ± 0.37	12.78 ± 1.03	101.1 ± 21.05 ^b^	0.48 ± 0.04	0.38 ± 0.14	0.60 ± 0.047
*O. leavegatus*	2.01 ± 0.03	4.56 ± 0.93	2.2 ± 0.08	13.28 ± 0.47	90.63 ± 15.3 ^c^	0.43 ± 0.04	0.33 ± 0.18	0.63 ± 0.20
*N. tenuis*	2.13 ± 0.11	4.83 ± 0.85	2.03 ± 0.59	13.34 ± 0.78	102.33 ± 18.6 ^b^	0.41 ± 0.046	0.38 ± 0.36	0.61 ± 0.06
*O. laevigatus* + *N. tenuis*	2.42 ± 0.28	4.33 ± 0.44	2.56 ± 0.26	13.97 ± 1.84	124.66 ± 8.17 ^a^	0.40 ± 0.05	0.32 ± 0.11	0.50 ± 0.30
Chemical insecticide	1.88 ± 0.17	5.23 ± 1.08	2.46 ± 0.49	12.13 ± 1.40	97.63 ± 15.5 ^bc^	0.51 ± 0.101	0.34 ± 0.20	0.51 ± 0.20

Different lowercase letters indicate statistically significant differences among treatments according to Tukey’s honestly significant difference (HSD) test at *p* < 0.05.

## Data Availability

The original contributions presented in this study are included in the article. Further inquiries can be directed to the corresponding author.
